# Editorial: The Molecular Mechanisms of Cyclic AMP in Regulation of Immunity and Tolerance

**DOI:** 10.3389/fimmu.2017.00076

**Published:** 2017-02-06

**Authors:** Josef Bodor

**Affiliations:** ^1^BIOCEV, Vestec, Czech Republic; ^2^Institute of Immunology, Johannes Gutenberg University, Mainz, Germany

**Keywords:** cyclic AMP, inducible cAMP early repressor, interleukin-2, CD28-responsive element, conventional CD4^+^ T cells, naturally occurring regulatory CD4^+^CD25^+^ T cells, graft-versus-host disease

**Editorial on the Research Topic**

**The Molecular Mechanisms of Cyclic AMP in Regulation of Immunity and Tolerance**

The inhibitory effects of the second messenger cyclic adenosine monophosphate (cAMP) on T-cell proliferation and effector functions have been originally reported by Gary Kammer in 1988 (1). At that time, little had been known about the mechanisms by which the inhibitory effects occur. The situation changed upon the discovery of the hallmark of cAMP responsive transcription—cAMP-responsive element binding (CREB) protein by Jim Hoeffler in Joel Habener’s lab at Massachusetts General Hospital (Boston, MA, USA) (2). However, it was still unclear how stimulation of the cAMP-dependent signaling pathway could exert an inhibitory effect on T-cell proliferation and effector functions. Soon after joining Joel Habener’s lab, I started to collaborate with Anna-Lena Spetz in Jack Strominger’s lab at the Dana-Farber Cancer Institute (Boston, MA, USA). Anna-Lena provided human thymus glands obtained from children undergoing corrective heart surgery and fractionated them over discontinuous Percoll gradients in order to separate medullary from cortical thymocytes. Our analyses confirmed that cAMP-dependent signaling is likely to play an important role during intrathymic T cell development since unexpectedly high intracellular levels of cAMP were formed in more mature medullary thymocytes in the presence of forskolin or prostaglandin E2. This ability to generate cAMP correlated with the expression of the potent transcriptional repressor ICER (inducible cAMP early repressor) and was retained by the majority of mature peripheral blood T lymphocytes (3). Induction of ICER was claimed to be exclusive to hypothalamic–pituitary–gonadal axis by Paolo Sassone-Corsi (4); however, our discovery of robust ICER expression in the human immune system came as a surprise (3). At the same time, Jeff Leiden demonstrated that thymocytes transgenic for a dominant negative isoform of CREB (DN CREB) were unable to phosphorylate the critical Ser at position 119 displayed after T cell activation. This functional defect resulted in a profound proliferative consequence characterized by markedly decreased interleukin-2 (IL-2) production (5). Clearly, a single point mutation of the critical Ser^119^ disabled activation domain in DN CREB acted to a similar extent as ICER, which lacks this activation domain entirely, and thus served as dominant negative repressor of IL-2 expression [note added in proof (3)]. Soon afterward, Ron Schwartz at the National Institutes of Health (Bethesda, MD, USA) redefined T cell anergy as failure to produce IL-2 *via* CREB/CREM-mediated transcriptional attenuation (6). Since ICER represents the only cAMP responsive subfamily of four isoforms (4), we concluded that at least part of inhibitory effects of cAMP on IL-2 production and T cell proliferation are due to exploitation of molecular mechanisms based on ICER induction and its high affinity binding at the CD28-responsive element (CD28RE) (7, 8). Since ICER does not possess a transactivation domain required for the recruitment of CREB binding protein (CBP)/p300, the binding of ICER to CD28RE and/or composite motifs containing CRE-like recognition sequences may also lead to uncoupling of CBP/p300, thus extinguishing IL-2 expression as well as expression of numerous other cytokines and chemokines driven by nuclear factor of activated T cells (NFAT) (9). Once T cell anergy had been redefined, Marc Gavin in Alexander Rudensky’s lab proposed another role for cAMP (and cAMP-induced ICER) in “anergy” of naturally occurring regulatory CD4^+^CD25^+^ T cells (nTregs) (10). He proposed that high intracellular levels of cAMP were maintained in nTregs by Foxp3-mediated repression of phosphodiesterase 3b, an enzyme responsible for cAMP degradation (11). This raised further questions, e.g., whether cAMP and ICER could play a role in nTreg cell-mediated suppression of IL-2 in CD4^+^ conventional T cells (Tcons). When I met Shimon Sakaguchi at the meeting in Boston, we discussed this possibility and he invited me to pursue this topic in his lab at Kyoto University in Japan. This resulted in a fruitful collaboration with Zoltan Fehervari who was already in Shimon’s lab at that time. During my stay in Shimon’s lab, Zoltan and myself proved that ICER/CREM in responder Tcons were definitively involved in nTreg cell-mediated suppression (12). Moreover, in discussion with Anjana Rao, we postulated the potential role of an inhibitory NFAT/ICER complex in Tcons as a means for induction of tolerance conveyed by nTreg cells. Meanwhile, Tobias Bopp in Edgar Schmitt’s lab at Uni-Mainz (Germany) reported cAMP as a key component of nTreg cell-mediated suppression (13). Tobias showed that transfer of the high intracellular cAMP levels accumulated in nTreg cells is critically involved in contact-dependent suppression of Tcons *via* intercellular gap junctions. To test this hypothesis, I joined Edgar Serfling’s lab in Wuerzburg (Germany), and in collaboration with Martin Vaeth, we performed confocal analyses of nTregs (14). Using two mouse models, we demonstrated that nTreg cells induced *via* cAMP a nuclear localization of ICER in Tcons and thereby inhibited their IL-2 synthesis. Ablation of nTreg cells using diphtheria toxin-mediated depletion of regulatory T-cells in DEpletion of REGulatory T cells mice resulted in cytosolic localization of ICER and increased IL-2 synthesis upon stimulation *in vivo* (14). Thus, upon stimulation with a CD28-superagonistic monoclonal antibody (15), direct contacts between nTreg cells and Tcons led to cAMP-dependent induction and nuclear accumulation of ICER and suppression of IL-2 synthesis in Tcons as hypothesized previously (14). We presumed that a close interaction of ICER and NFAT bound to the IL-2 promoter in Tcons is of vital importance for the nTreg cell-mediated suppression. Therefore, we attempted to crystallize the NFAT/ICER complex in cooperation with Caroline Kisker at Uni-Wuerzburg, and soon, we obtained crystals. Unfortunately, their analyses showed that despite expectations they did not contain all the components in the complex. Meanwhile, Anjana Rao together with Lin Chen crystallized the NFAT/Foxp3 complex and claimed this complex to be central to tolerance conveyed by nTreg cells (16). However, this work was flawed by two major problems: (i) they did not use Foxp3 but Foxp2 (a gene with function quite distinct from Foxp3) and (ii) nTreg cells do not translocate NFAT efficiently to the nucleus, a finding reported by us (14) and others (17). In conclusion, convincing proof of the existence of a molecular complex central to immune tolerance (NFAT/ICER or NFAT/FoxP3) remains elusive and awaits further investigation.

## Author Contributions

JB contributed to this editorial in its entirety.

## Conflict of Interest Statement

The author declares that the research was conducted in the absence of any commercial or financial relationships that could be construed as a potential conflict of interest.
